# The Challenge to Decide between Pulmonary Hypertension Due to Chronic Lung Disease and PAH with Chronic Lung Disease

**DOI:** 10.3390/diagnostics11020311

**Published:** 2021-02-15

**Authors:** Horst Olschewski

**Affiliations:** 1 Department of Internal Medicine, Division of Pulmonology, Medical University of Graz, Auenbruggerplatz 15, A-8036 Graz, Austria; horst.olschewski@medunigraz.at; 2 Ludwig Boltzmann Institute for Lung Vascular Research, A-8010 Graz, Austria

**Keywords:** pulmonary hypertension, pulmonary arterial hypertension, PAH drugs, right heart catheter investigation, chronic obstructive lung disease

## Abstract

Chronic lung diseases are strongly associated with pulmonary hypertension (PH), and even mildly elevated pulmonary arterial pressures are associated with increased mortality. Chronic obstructive pulmonary disease (COPD) is the most common chronic lung disease, but few of these patients develop severe PH. Not all these pulmonary pressure elevations are due to COPD, although patients with severe PH due to COPD may represent the largest subgroup within patients with COPD and severe PH. There are also patients with left heart disease (group 2), chronic thromboembolic disease (group 4, CTEPH) and pulmonary arterial hypertension (group 1, PAH) who suffer from COPD or another chronic lung disease as co-morbidity. Because therapeutic consequences very much depend on the cause of pulmonary hypertension, it is important to complete the diagnostic procedures and to decide on the main cause of PH before any decision on PAH drugs is made. The World Symposia on Pulmonary Hypertension (WSPH) have provided guidance for these important decisions. Group 2 PH or complex developmental diseases with elevated postcapillary pressures are relatively easy to identify by means of elevated pulmonary arterial wedge pressures. Group 4 PH can be identified or excluded by perfusion lung scans in combination with chest CT. Group 1 PAH and Group 3 PH, although having quite different disease profiles, may be difficult to discern sometimes. The sixth WSPH suggests that severe pulmonary hypertension in combination with mild impairment in the pulmonary function test (FEV1 > 60 and FVC > 60%), mild parenchymal abnormalities in the high-resolution CT of the chest, and circulatory limitation in the cardiopulmonary exercise test speak in favor of Group 1 PAH. These patients are candidates for PAH therapy. If the patient suffers from group 3 PH, the only possible indication for PAH therapy is severe pulmonary hypertension (mPAP ≥ 35 mmHg or mPAP between 25 and 35 mmHg together with very low cardiac index (CI) < 2.0 L/min/m^2^), which can only be derived invasively. Right heart catheter investigation has been established nearly 100 years ago, but there are many important details to consider when reading pulmonary pressures in spontaneously breathing patients with severe lung disease. It is important that such diagnostic procedures and the therapeutic decisions are made in expert centers for both pulmonary hypertension and chronic lung disease.

## 1. Historical Introduction

In the year 1898, Dr. Ernst Romberg, one of our pioneers, stated that pulmonary hypertension, in chronic heart or lung diseases, was well known and not at all interesting for the clinician scientist. Pulmonary hypertension was interesting only when it developed without such underlying conditions [1]. This tells us that more than a century ago, chronic diseases of the lung, at that time mostly tuberculosis and emphysema, were well established as common causes of pulmonary hypertension.

In line with this, the first WHO Symposium on Pulmonary Hypertension, 1973, made a clear cut between primary pulmonary hypertension (PPH) and secondary pulmonary hypertension. This appeared even easier than the definition of pulmonary hypertension (PH) itself. After long discussions, the PH threshold was set at a mean pulmonary arterial pressure (mPAP) of 25 mmHg at rest, although it was well known, that in healthy subjects, mPAP rarely exceeds 20 mmHg. Exercise appeared less exciting at that time, as there was a strong belief, that mPAP would not increase much during exercise, even if cardiac output increased 3-5-fold. Accordingly, the threshold for mPAP during exercise was set just 5 mmHg higher, at 30 mmHg. Today, there is clear evidence that setting a threshold for resting mPAP at 25 mmHg was not scientifically justified [2,3] and that the assumption of nearly constant mPAP during exercise was simply wrong [3,4]. But what happened with PPH vs. PH due to lung disease?

At the second WHO Symposium in Evian, 1998, experts again discussed the classification of PH. Once more, there was a broad consensus about the causal relationship between diseases of the heart and PH (group 2) and between diseases of the lung and PH (group 3) and that recurrent pulmonary thromboembolism was the cause of chronic thromboembolic pulmonary hypertension (CTEPH, group 4). However, there was a tough discussion how to classify collagen vascular diseases, appetite suppressant drugs, small left-to-right shunts, portal hypertension and HIV infection, which were all known or at least believed to respond to prostacyclin, like PPH did [5,6]. In addition, some of these diseases, e.g., small atrial septal defects, apparently needed a genetic predisposition for PH, similar to PPH. It took just 2 more years until the first causal mutation underlying PPH was discovered [7,8], but the underlying genetic causes for the majority of the other forms of PH has remained a major field of research. And this does not only include PAH but all PH classes! 

Finally, it was decided to define a new class, called pulmonary arterial hypertension (PAH, group 1), including PPH (today idiopathic and heritable PAH and responders to high-dose calcium channel blockers) and some other forms of PH, with the strong belief that they all shared similar genetic predispositions, pathologic mechanisms and therapy responses. This also meant that group 2–5 PH patients were deemed to be different in all three aspects. 

Particularly in the case of group 3 PH, it seemed obvious that a genetic predisposition was not mandatory. The known pathologic mechanisms were meant to be strong enough and very different from PAH, and the therapy response was even opposite. Such patients did not profit from high-dose calcium channel blockers nor from prostacyclin. Today, there are much more data available, but the principles of the PH classification have remained the same, resulting in quite different therapeutic recommendations for PAH and group 3 PH. 

## 2. Need for Biomarkers

Only if there is a known mutation causing PAH, or the patient’s family is strongly affected by PAH, we can be quite sure that this patient suffers from Group 1 PAH. However, such mutations are very rare and diagnostics are still too expensive to apply them on a large scale. There is an unmet need for biomarkers allowing the differential diagnosis between PAH and PH due to chronic lung disease. Even in explanted lungs or biopsy samples, there are no specific changes, indicating PAH, as compared to other forms of PH [9]. Historically, chronic depolarization of pulmonary arterial smooth muscle cells (PASMC) was described as a typical feature of PPH, causing calcium entry, constriction and proliferation. More than 20 years ago, the loss of a voltage-gated potassium channel, explaining this depolarization, was believed to be specific for PPH [10]. Furthermore, there is an inactivation of the TASK-1 potassium channel [11] depending on the tyrosine kinase c-src [12] and an upregulation of the chloride channel TMEM 16A [13] which both can explain the depolarization of IPAH PASMC, however, there is no evidence that this can help to differentiate between group 1 and group 3 disease. 

Microarray-based analysis of explanted lungs or small pulmonary arterial vessels from patients with PH due to PAH, COPD and interstitial lung disease showed striking changes as compared to heathy controls [14], however, no specific signature for PAH as compared to group 3 PH. When we analyzed the genetic profiles of small pulmonary arteries from PH due to COPD vs. interstitial lung disease, we were surprised to find quite different profiles in the gene expression [15]. This suggests that we are far away from a blood-derived biomarker that helps us in the differential diagnosis between group 1 and group 3 PH. 

Whole genome sequencing of deep-phenotyped patients with group 1 PAH with chronic lung disease vs. group 3 PH due to lung disease could provide more insight in the specific genetic differences and is urgently warranted. 

## 3. Current Therapy Recommendations for Group 3 PH as Compared to PAH

Although it is not the main focus of this review, I will briefly touch on therapy. Indeed, the therapeutic consequences are the main reason to care so much for the distinction between group 1 PAH and group 3 PH. 

During the last two decades, for PAH more than 10 targeted medications have been approved after well conducted randomized controlled studies. Current guidelines recommend applying PAH targeted medication according to a therapy algorithm that is strongly oriented to the patients’ risk stratification [16]. Non-responders, the vast majority of PAH patients, receive initial oral combination therapy. If they benefit, they keep going on, if they deteriorate or do not benefit enough, they proceed to triple therapy including an intravenous prostanoid. 

In contrast, for group 3 PH patients, PAH medication is not recommended [17]. Guidelines rather recommend optimized treatment of the underlying disease, long-term oxygen therapy, eventually home ventilation and lung transplantation. Only if there are signs and symptoms of circulatory failure due to severe PH, may individualized PAH therapy be indicated in PH expert centers [17,18]. 

The reason for the negative recommendation for group 3 PH is, that all randomized controlled trials with any PAH drugs have failed. This relates to both COPD [19,20] and interstitial lung diseases such as idiopathic pulmonary fibrosis (IPF) [21,22,23,24]. In IPF, there might be some positive effects in certain secondary endpoints with sildenafil without [23] or with targeted IPF medication [25,26], but still: the primary endpoints remained negative.

**Figure 1:**
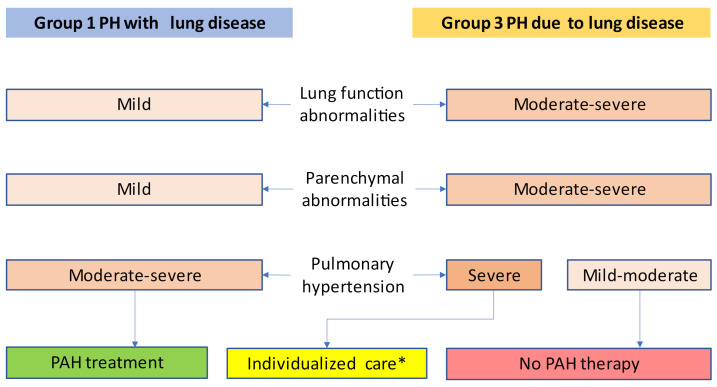
Therapeutic consequences derived from decisions between group 1 PH vs. group 3 PH and severe vs. non-severe pulmonary hypertension. * Therapy decision reserved to expert centers for both chronic lung diseases and pulmonary hypertension. Adapted from Nathan et al. [18].

There were, however, some positive experiences published as case reports and case series, describing favorable effects of PAH medication mostly in the subgroup of patients with the most severe pulmonary hypertension [27,28,29,30]. The important notion is that the beneficial effects of PAH drugs in these uncontrolled observations were found in patients with decompensated right heart failure or severe pulmonary hypertension and not in mild-to-moderate PH. This is the reason why the severity of pulmonary hypertension is so important in group 3 PH (Figure 1).

The current PH guidelines were launched by ESC and ERS in 2015 [17] and they have been commended by the Cologne Consensus Conference (CCC) [31,32], an expert conference within the German-speaking countries. In addition, the Proceedings from the sixth World Symposium on PH (WSPH) in Nice, 2018, have been published, providing the newest evidence and expert opinion on a world-wide scale [16,18].

## 4. Relevance of Elevated Pulmonary Arterial Pressure in COPD

### Impact of Pulmonary Arterial Pressure on COPD Prognosis

The first study, addressing the impact of pulmonary arterial pressure on survival in COPD patients, originated from Emanuel Weitzenblum’s group in Strasbourg, in the year 1980, showing a significant association between PAP elevation and increased mortality [33]. Many studies from the same and other groups confirmed this result. Interestingly, the threshold between a favorable and a poor prognosis in all studies was around 19–20 mmHg i.e., in the upper normal range of pulmonary arterial pressure.

The ASPIRE registry was the first to describe a cohort of patients with chronic lung disease that had been admitted to a PH clinic for a possible PH therapy. It turned out that this subgroup of PH patients had the worst prognosis as compared to all other PH groups [34] and in the subgroup of COPD patients, severely increased mean pulmonary arterial pressure (mPAP) >40 mmHg was associated with an even worse prognosis than mPAP between 25 and 40 mmHg [30]. Higher age and WHO functional class, lower DLCO and SVO_2_ were independent risk factors for a poor prognosis. Unfortunately, pulmonary vascular resistance (PVR), in the multivariate analysis, was not tested for its prognostic impact. However, in the univariate analysis, PVR was a stronger risk factor for mortality than PAP itself.

## 5. Exacerbation

In a small but seminal study, PAP as measured by RHC, and arterial pCO_2_ were independent predictors of severe COPD exacerbation [35]. Interestingly, again the threshold for an increased exacerbation rate was again at a PAP of 19 mmHg. More recently, a large prospective evaluation of the COPDgene and the ECLIPSE cohort, including about 5000 COPD patients in GOLD Stage 2 und 3, found that an elevated pulmonary arterial/aortic diameter-ratio (PA/AO-ratio), as derived from thin-slice CT, was the strongest baseline predictor of exacerbation [36]. PA/AO >1 was associated with a nearly five-fold exacerbation risk (*p* < 0.001). As a drawback of the study, RHC was not available. However, it is well known that the PA/AO-ratio is strongly associated with mPAP [37]. This supports the early findings from Strasbourg, that even a mild PAP elevation is associated with an increased exacerbation rate.

There is another piece of evidence: the CHAMPION trial investigated the long-term association between PAP and hospitalization in left heart disease by means of the CardioMems^®^ device [38]. It showed that recognition of an increase in PAP, resulting in optimized management with diuretics, prevents hospitalization events. In the CHAMPION cohort, all patients suffered from left heart disease, but 34% of them also suffered from COPD. The COPD subgroup profited as much from the CardioMems^®^ device as the other patients. Interestingly, in the COPD subgroup, most hospitalizations were labelled as “COPD exacerbation” [39]. This suggests that in COPD patients with heart failure, PAP increase predicts COPD exacerbation. It also suggests that these “COPD exacerbations” can be prevented by optimized management with diuretics.

## 6. How Common Is PH in COPD?

### 6.1. Mild to Moderate PH

About 90% of severe emphysema patients present with mPAP >20 mmHg and 50% of severe emphysema patients present with mPAP >25 mmHg [40]. This suggests that mild to moderate PH is very common among COPD patients, and therefore also among the general population.

### 6.2. Severe PH

In 2005, there were two instrumental papers from France on the question of how common severe PH in COPD is. One was a long-term systematic right heart catheter-based investigation from Strasbourg. It showed that out of 998 COPD patients, investigated in a stable phase of their COPD, just 2.7% suffered from severe PH, defined as a mean PAP >40 mmHg. If potential other causes, such as lung embolism, collagen vascular disease, and left heart disease, were excluded, the rate of severe PH further decreased to 1.1% [41]. These patients were characterized by a low DLCO and relatively low pCO_2_. The other study analyzed a COPD cohort of 215 patients admitted for lung volume reduction surgery or transplantation [42]. Cluster analysis provided four different clusters, one of which was characterized by exceptionally high mPAP, but moderate ventilatory obstruction, and relatively low arterial pCO_2_. In this cluster, mPAP was around 40 mmHg, paO2 and paCO_2_ were 46 and 40 mmHg, respectively, and FEV_1_ was 48% predicted, the best FEV_1_ out of all clusters.

This suggests that severe PH in COPD exists, but that it is only partly due to COPD, and that these patients have relatively well preserved ventilatory lung function. In a recent perspective on this topic, this type of COPD was discussed as the “pulmonary vascular phenotype” [43].

### 6.3. From Relations to Absolute Patient Numbers

It is difficult to imagine what it means when a percentage of a percentage of people is affected. Therefore, I will use an example which is based on the number of patients with moderate-to-severe COPD in the German population. The Copenhagen Heart&Lung Study [44] found a prevalence of 0.7% for stage III + IV COPD among the general population >40 yr (471/61.650). The German population with the same age distribution comprises 47 million people, and the corresponding number of stage III + IV COPD cases would be 360,000. If just 1.1% of these have mPAP >40 mmHg due to their COPD, this is nearly 4000 patients. This can be compared with the PAH prevalence from the COMPERA registry, that mainly enrolled patients from Germany, and found fewer than 2000 PAH patients [45]. This is less than half the number of severe PH due to moderate-to-severe COPD. Of course, there are many more COPD patients with less severe COPD and mPAP between 35 and 40 mmHg, and in many more patients, COPD is not the only cause of pulmonary hypertension. This would drive the ratio of severe COPD PH / PAH further up. However, the true number of PAH patients in Germany may also be underestimated, because participation in the COMPERA registry was not mandatory for German PH centers.

Nevertheless, this calculation suggests that severe PH due to COPD may be more prevalent than all forms of PAH together.

## 7. What Are the Factors Causing PAP Increase in Chronic Lung Disease?

There are many mechanisms contributing to elevated mPAP in chronic lung disease. To explain this, I will use a simple hemodynamic approach starting from the relationship of pressures and flows in the pulmonary circulation. The hemodynamic factors contributing to PAP are defined by pulmonary venous pressure (PAWP), pulmonary vascular resistance (PVR), and cardiac output (CO), simply because:

PAP = PAWP + PVR × CO (1)

However, there are several pitfalls, which have been addressed in our previous review [46] and in an recent ERS Task Force report [4]. The major issues are setting the zero level to the right point and the method of reading the pressures.

### 7.1. Zero Level

The fifth WSPH suggested to place the zero level on the left atrial level [47], i.e., according to the mean sagittal thoracic diameter [48]. This is particularly important in COPD, because emphysema may change the thoracic dimensions, lifting the left atrium more than usual above the catheter table level. Therefore, it is important to make sure that the zero level is set at the mid-thoracic level and not e.g., 10 cm above table.

### 7.2. Intrathoracic Pressure

Unfortunately, intrathoracic pressure adds to all PAP and PAWP readings. In non-obstructive patients, this factor is mostly small and negligible, however, in severe COPD, particularly during exercise, it can exceed +10 mmHg during end-expiration [46]. Surprisingly, the sixth WSPH suggested end-expiratory measurement of intrathoracic pressures. However, this is based on the normal physiology, where at end-expiration, for a little moment, there is no active intrathoracic pressure generation, leaving a small time period to make the reading. In patients with significant ventilatory obstruction, however, there is no such time period. At end-expiration there is still a positive ventilatory pressure, and immediately afterwards there is a deep inspiratory dip, sometimes shifting PAP into the negative range. If a breathing maneuver is applied, the pressure readings become absolutely unpredictable. Therefore, such maneuvers are not recommended [47]. If end-expiratory reading is difficult at rest, it is absolutely impossible during exercise. This speaks in favor of employing a floating digital average over several complete respiratory cycles at both rest and exercise for patients with and without lung disease [4,46]. Even if this is done, the loss of elasticity of the lungs in COPD causes increased average intrathoracic pressures and thereby an increase in both PAP and PAWP, which is independent of PVR and cardiac function. The opposite changes are seen in restrictive lung diseases, where RAP and PAWP can be near to zero.

### 7.3. Cardiac Output (CO)

In chronic lung diseases, CO is often normal and even in the upper normal range. This may have several reasons but one important cause is hypoxic vasodilatation of the systemic arteries which causes systemic vascular resistance (SVR) to decrease and a secondary activation of the arterial baroreflex. Indeed, the hypoxia-mediated effects in systemic arteries are opposite to the pulmonary arteries [49].

### 7.4. Pulmonary Arterial Wedge Pressure (PAWP)

PAWP may be elevated due to left heart failure (systolic or diastolic) or due to air trapping as discussed above. Heart failure is strongly associated with old age and systemic hypertension as well as with sleep apnea syndrome and obesity. Heart failure and air trapping are mostly easy to distinguish from each other, because air trapping causes deep pressure swings with respiration. However, sometimes it is difficult to exactly discern the two components, particularly during exercise [4].

### 7.5. Pulmonary Vascular Resistance

The mechanisms causing an increased vascular resistance of the pulmonary vessels have recently been reviewed [50]. PVR can be elevated by rarefication of the pulmonary vessels or by constrictive remodeling. Vascular rarefication is difficult to quantify, although it is known that the number of vessels is often reduced due to emphysema or due to lung fibrosis. For PVR, the countable medium-sized vessels are less important than the small pulmonary arteries but these cannot be counted so easily. In addition, in chronic lung diseases, there can be right-to-left shunt blood flow through dilated pulmonary arterioles/megacapillaries, reducing PVR despite reduced vessel numbers.

In contrast, there is much more evidence for hypoxic pulmonary vasoconstriction or, if it persists for long time, hypoxic remodeling of the small pulmonary arteries. This has been very well documented over the last 120 years [50]. We have recently shown that p22phox, an essential regulator protein from NADPH oxidase, is associated with the constrictive remodeling in the hypoxic mouse model [51]. We have further shown that p22phox is regulated in explant lungs from end-stage COPD patients: preserved p22phox was associated with better-than-average preserved ventilation/perfusion-matching, but it was also associated with low DLCO and high mPAP [51]. This suggests that genetic regulation contributes to the vascular remodeling in COPD patients.

Mild or moderate PH is often caused by just CO- and intrathoracic pressure elevation. This may explain that PAH medication is futile. However, severe PH is only possible if there is a severe constrictive remodeling of the pulmonary arteries and this may be the right substrate for PAH therapies. The best indicator of such remodeling is a severe elevation of PVR.

## 8. Cause of Severe Pulmonary Arterial Remodeling in Smokers

Autopsy studies have shown neo-muscularized and stiffened small pulmonary arteries in COPD lungs. This is mimicked by chronic hypoxia in the animal model and worsened by VEGF antagonists causing endothelial dysfunction [52,53]. The tool used for most of these experiments was SU5416 (Sugen), a tyrosine kinase inhibitor. A recent investigation found that Sugen-induced emphysema was associated with suppression of hepatocyte growth factor and can be rescued by HIF-2α [54], a protein that mediates hypoxic pulmonary vasoconstriction and remodeling [55,56]. This suggests that these mechanisms are much more complex than previously thought.

Smokers may also develop pulmonary arterial remodeling, even without significant FEV_1_ decrease or hypoxia [57]. This is mimicked in the smoking mouse model [58]. After 6 months, the smoking mouse develops pulmonary arterial remodeling and after 8 months also lung emphysema. Inducible NO synthase (iNOS) lacking mice were protected from both vascular remodeling and emphysema. In this mouse model, an iNOS inhibitor reversed both vascular remodeling and emphysema. This underlines the importance of smoking-induced inflammation in the pathogenesis of both pulmonary arterial remodeling and emphysema.

## 9. Clinical Classification of Lung Diseases with PH (Nizza Group 3 vs. Group 1)

**Figure 2:**
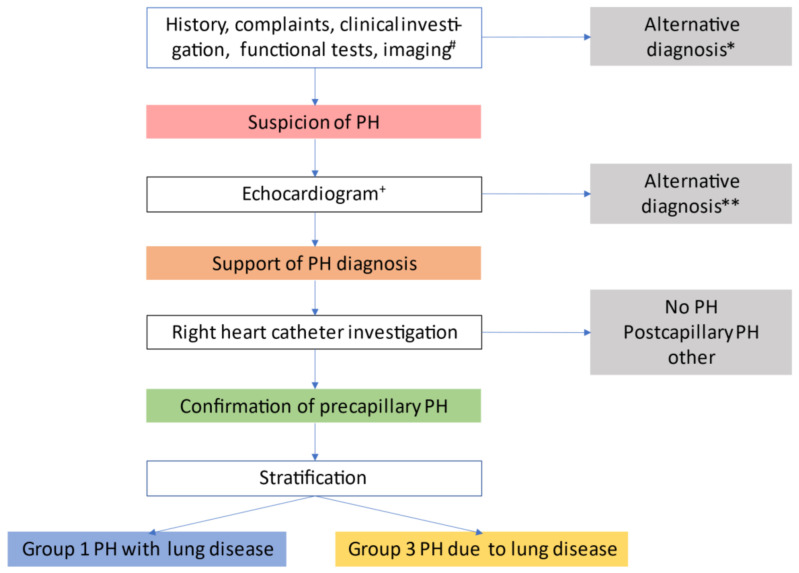
Evaluation of pulmonary hypertension in chronic lung disease. ^#^ suggestive findings include 1) symptoms and signs (dyspnoea out of proportion, loud P2, signs of right heart failure, right axis deviation on ECG, elevated natriuretic peptide levels); 2) pulmonary function test abnormalities e.g., DLCO < 40%, elevated %FVC/%DLCO-ratio (low KCO); 3) exercise test findings (including decreased distance, decreased arterial oxygen saturation or increased Borg rating on 6 min walk test and decreased circulatory reserve, preserved ventilatory reserve on cardiopulmonary exercise testing); and 4) imaging findings (extent of lung disease, enlarged pulmonary arterial trunk on chest x-ray, increased PA/AO-ratio on CT). *Uncontrolled systemic hypertension, bradycardia, arrhythmia, myocardial ischemia, obesity, thyroid disorder, depression, acute/subacute pulmonary embolism, exacerbation of lung disease. ** Systolic or severe diastolic myocardial failure, valvular heart disease, pericardial disease, congenital heart disease. ^+^ signs supporting the diagnosis of PH include elevated SPAP and signs of right ventricular dysfunction. Signs supporting the diagnosis of severe PH include reduced TAPSE/SPAP-ratio [59]. Adapted from Nathan et al. [18].

The proceedings of the sixth WSPH suggested an algorithm covering the steps from clinical suspicion over echocardiographic support to PH diagnosis by RHC to stratification for group 1 vs. 3 PH [18]. Figure 2 illustrates the work-up of patients. This may guide physicians through the identification process of PH, defining the correct diagnosis and pinpointing those group 3 patients who might benefit from PAH therapy, according to Figure 1.

Importantly, patients with a group 1 PH (PAH) and a concomitant lung disease may be treated like PAH patients, who do not suffer from such a co-morbidity [17,18]. This recommendation is based on subgroup analysis (unpublished) from randomized controlled PAH studies, where patients with mild to moderate pulmonary comorbidities had been enrolled [60,61,62]. These patients had no significantly different therapy responses and adverse effects than the other study patients. Despite this, there are good reasons to assume a higher risk for adverse effects in PAH with chronic lung disease, simply because these patients may also develop exacerbations of their lung disease. In addition, as all approved PAH medications are strong vasodilators, the relaxation of constricted pulmonary vessels may attenuate hypoxic pulmonary vasoconstriction and thus worsen the oxygenation, particularly in periods of COPD exacerbation. The second potential adverse mechanism of vasodilators is edema formation, particularly in the elderly.

The treatment recommendations for group 3 PH are different from PAH treatment recommendations. For the vast majority of group 3 PH patients, PAH medication is not recommended [17,18]. Only for those few with severe PH, PAH medication should be considered. For this reason, the two most important decisions to be made are between group 1 vs. 3 PH and between severe vs. non-severe PH.

The proceedings of the sixth WSPH suggested the criteria listed in the Table 1 to decide between group 1 and 3 PH.

## 10. Defining Severe PH

Circulatory failure and severe PH are difficult to define. The international expert conferences made a reasonable suggestion, although purely based on expert opinion (Table 2):

It is also noteworthy that these criteria do not apply to PAH patients. This is another reason to clearly distinguish between group 1 and 3 PH.

According to the ESC/ERS PH guidelines, patients with severe COPD PH or right ventricular failure are candidates for individual therapy decisions on PAH therapies by expert centers [17,18]. Although in such patients, PAH drugs may have similar hemodynamic effects as in group 1 PAH with a concomitant lung disease, they are even more prone to adverse effects because their lung disease is more severe.

## 11. Conclusions

The combination of COPD and PH is often but not always caused by the effects of the chronic lung disease on the pulmonary circulation. Unfortunately, there are no reliable biomarkers or singular tests to stratify these patients to Group 1,2,3,4, and 5 PH. In chronic lung diseases, even a mild PAP elevation predicts a poor prognosis, however, in most cases, targeted PAH therapy is not indicated. If either COPD causes severe PH, or COPD is a concomitant disease in a PAH patient, PAH therapy may be indicated. However, these patients, due to their co-morbidity, are more prone to adverse effects, particularly exacerbations, and therapy decisions are more difficult because also PAH medication may exert more adverse events than in PAH patients without co-morbidities. Therefore, such patients should always be referred to PH expert centers for individual therapy decisions.

## Figures and Tables

**Table 1:** Criteria for Group 3 PH.

Criteria for Group 3 PH:
FEV_1_ <60% or VC < 70%
Moderate or severe parenchymal abnormalities in lung HRCT
Features of exhausted ventilatory but not circulatory reserve in the cardiopulmonary exercise test.

FEV_1_, one-second expiratory capacity in % predicted; VC, vital capacity in % predicted; DLCO, diffusion capacity for carbon monoxide in percent predicted. Adapted from Nathan et al. [18].

**Table 2:** Criteria for severe PH in patients with chronic lung disease.

Criteria for Severe PH
Resting mPAP >35 mmHg
mPAP ≥ 25 mmHg with CI <2.0 L/min/m^2^)

mPAP, mean pulmonary arterial pressure, as measured by right heart catheter. CI, cardiac index. Adapted from Nathan et al. [18].
